# Effects of Moisture Infiltration on Interfacial Characteristics of Fiber Asphalt Mastic-Aggregate and the Cracking Resistance of Mixture

**DOI:** 10.3390/ma18010053

**Published:** 2024-12-26

**Authors:** Keke Lou, Silin Jia, Peng Xiao, Haochen Wu, Yuhao Wu

**Affiliations:** 1 College of Civil Science and Engineering, Yangzhou University, Yangzhou 225100, China; lkkyzu@163.com (K.L.); 18994185805@163.com (S.J.); 17715878456@163.com (H.W.); wyh19980402@163.com (Y.W.); 2 Research Center for Basalt Fiber Composite Construction Materials, Yangzhou 225127, China

**Keywords:** asphalt mixture, moisture infiltration, fiber, interfacial strength, cracking resistance

## Abstract

The interfacial properties of fiber asphalt aggregate and the cracking resistance of asphalt mixture are directly affected by moisture infiltration. In order to investigate the correlation between interfacial properties and immersion stability of asphalt mixture, three different types of fiber, including basalt fiber (BF), glass fiber (GF), and polyester fiber (PF); five types of fiber contents (0.1% to 0.5% by mass of the mixtures); and two types of aggregates (basalt and limestone) were selected. Experimental methods such as the Bond Strength Test (BBS), Disk-Shaped Compact Tension test (DCT), and interfacial image processing were used in order to assess the strength of interfacial interaction and resistance to cracking under both dry and wet conditions. The results showed that the addition of fibers could enhance fiber asphalt mastic-aggregate interfacial strength; under the influence of moisture infiltration, the interfacial strength showed a significant downward trend. In the process of fiber content increasing from 0.1% to 0.5%, the peak load and fracture energy of fiber asphalt mixtures were first increased and then decreased. The interface between asphalt mastic and aggregate is easier to spalling after being subjected to moisture infiltration, resulting in a decrease in cracking resistance. Compared with the dry environment, after moisture infiltration, the correlation index between interfacial strength and fracture energy is much higher than other influencing factors. The interfacial strength is still an important factor affecting the fracture energy. These findings provide valuable insights for the design and application of more durable asphalt pavement.

## 1. Introduction

Asphalt mixture is widely utilized in road construction because of its advantages, such as driving comfort. Throughout the service life of asphalt pavement, continuous loading can cause various types of damage, while precipitation and prolonged exposure to moisture can accelerate the deterioration of these mixtures [1,2]. Water damage poses a significant threat to the durability and performance of the asphalt mixture, as moisture infiltration into the asphalt matrix leads to a range of detrimental effects. These include the detachment of asphalt binders from aggregates, reduced load-bearing capacity, and premature structural failure [3]. Such issues not only compromise the integrity of roadways but also increase maintenance costs and shorten their lifespan [4], attracting considerable attention from researchers and engineers worldwide. Many researchers have extensively explored the mechanisms of water damage, yielding valuable insights. Researchers [5,6,7,8] employed various techniques to study the microscopic adhesion between aggregates and asphalt and to assess the water stability of different asphalt-aggregate combinations. Additionally, Kai Zhang [9] employed low-field nuclear magnetic resonance technology to observe the pore structure parameters in asphalt mixtures under cyclic loading, noting that under the action of cyclic load and dynamic water erosion, some micropores expand and crack and form larger pores.

Fiber-reinforced asphalt mixtures present an effective approach to enhancing the mechanical and overall performance of asphalt materials [10]. Recent studies indicate that the incorporation of fibers into asphalt mixtures can significantly improve tensile strength, reduce permanent deformation, and improve resistance to thermal cracking. Various types of fibers, including synthetic and natural fibers, have been used to improve the viscoelastic properties of asphalt, offering a promising solution to the challenges posed by water damage. Carlos J. Slebi-Acevedo [11] suggests that the addition of fibers can improve fatigue resistance, reduce permanent deformation, and increase stiffness. Fibers reinforce hot mix asphalt through a three-dimensional network, increasing the adhesion within the mixture. L.M.G. Klinsky [12] demonstrated that the use of polypropylene and aramid fibers significantly enhanced the mechanical properties of conventional hot mix asphalt, enhancing its resistance to water damage and improving its ability to withstand rutting and permanent deformation. This results in better fatigue cracking resistance and a greater capacity to resist reflective cracking. Jie Wu [13] identified the asphalt adsorption effect and the three-dimensional spatial network structure as the primary reinforcement mechanisms of natural fibers, which effectively suppress rutting and cracking in asphalt pavements. Akihiro Sato [14] used optical microscopy and electron microscopy to characterize the morphology of the cellulose nanofibers and evaluated the rheological and mechanical properties of the fibers. Additionally, Hayder Kamil Shanbara’s [15] research showed that using glass and hemp fibers as reinforcing materials significantly improved the water sensitivity of cold-mix asphalt, thereby enhancing its resistance to surface cracking. Extensive research has revealed that the stability of the bond between aggregates and asphalt is the key to the formation of mixture performance, where poor interactions can lead to issues such as pavement cracking, water damage, and permanent deformation. Molecular dynamics simulations have been employed to understand the interactions between asphalt and aggregates at the nanoscale [16,17]. In terms of aggregate characteristics affecting interface interactions, Jizhe Zhang [18] and colleagues conducted peel tests on four different aggregates and two types of asphalt, exploring how the mineral composition of aggregates influences water absorption. Their findings indicated that the mineral composition affects the aggregate’s sensitivity to moisture. Curtis et al. [19,20] found that various components within asphalt can influence the bond strength at the interface. Several well-established methods exist for evaluating the characteristics of the asphalt binder-aggregate interface, including mechanical tensile testing [21,22], asphalt stripping tests [23,24,25], surface energy methods [26], and adhesion fatigue testing [27].

In recent years, the crack resistance of fiber-reinforced asphalt mixtures has garnered vital attention. Research indicates that the incorporation of fibers not only delays crack formation but also enhances the overall toughness of asphalt mixtures. A comprehensive understanding of how environmental conditions interact with the performance of fiber-reinforced mixtures is essential for developing strategies to improve their cracking performance under various service conditions. Md Nafiur Rahman [28] evaluated the crack resistance of polyethylene aramid and nylon fiber-reinforced asphalt mastic by conducting two crack mouth opening displacement-controlled experiments, revealing that nylon fiber exhibits superior crack resistance. Moreover, the inclusion of sugarcane bagasse fibers can enhance the high-temperature stability and low-temperature crack resistance of asphalt mixtures, achieving performance comparable to lignin fiber-reinforced mixtures [29]. The single-edge notched three-point bending tests demonstrated that polypropylene fibers increase the strength and fracture toughness of asphalt mastic, with crack bridging and fiber pullout identified as the two primary toughening mechanisms [30]. Yao Zhang [31] employed X-ray computed tomography, discrete element simulation techniques, digital image processing, and low- and medium-temperature cracking tests to obtain the optimal fiber length distribution so as to enhance crack resistance. Qin Tang [32] investigated the effects of different fiber types, fiber contents, and aggregate types on interface properties. The results indicated a strong correlation between the interface interaction strength of fiber-reinforced asphalt mastic and the fracture energy and peak crack mouth opening displacement of the asphalt mixtures.

**Figure 1:**
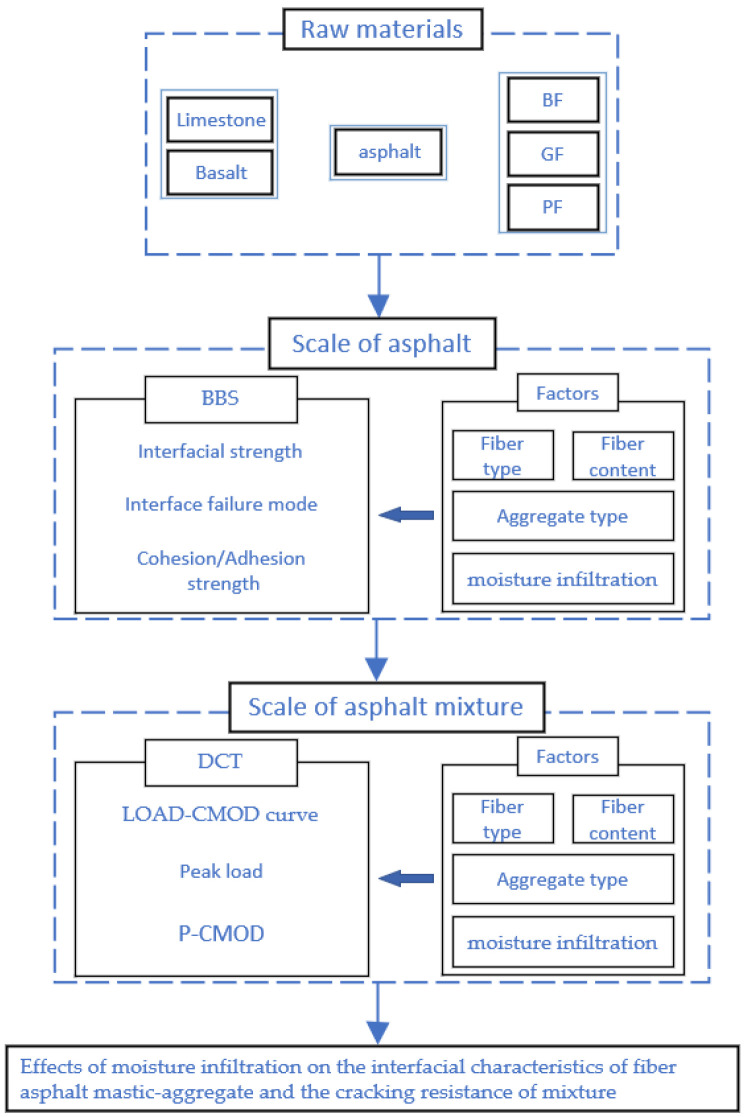
Flowchart of this study.

Currently, research on the interfacial and cracking performance of fiber-reinforced asphalt mixtures is limited, particularly regarding the interfacial characteristics and cracking resistance of fiber asphalt mastic-aggregate in a water environment. Therefore, it is crucial to conduct in-depth studies on the influence of moisture infiltration on these properties. The flow chart for this study is shown in Figure 1. By investigating the interaction between moisture infiltration and the properties of the mixture, this study aims to provide valuable insights for the design and application of more durable asphalt pavement.

## 2. Materials and Methods

### 2.1. Raw Materials

**Figure 2:**
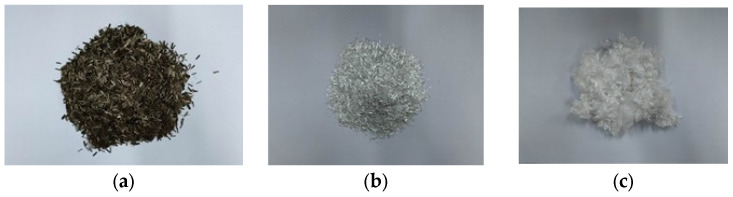
Macroscopic; (**a**) BF; (**b**) GF; and (**c**) PF.

SBS-modified asphalt was used in this study. The tests were conducted in accordance with the standard [33,34]. The penetration (25 °C, 0.1 mm) is 51.8, the ductility (5 cm/min, 5 °C) is 30 cm, and the softening point is 77 °C, respectively. The coarse aggregate was made of limestone and basalt. Three types of fiber were used in this experiment, including basalt fiber, glass fiber, and polyester fiber, and the surface morphology of the three kinds of fiber is shown in Figure 2, and the technical parameters are shown in Table 1. The basalt fiber is hereinafter referred to as BF, glass fiber as GF, and polyester fiber as PF.

### 2.2. Preparation for Fiber-Reinforced Asphalt Mixture and Asphalt Binder

**Figure 3:**
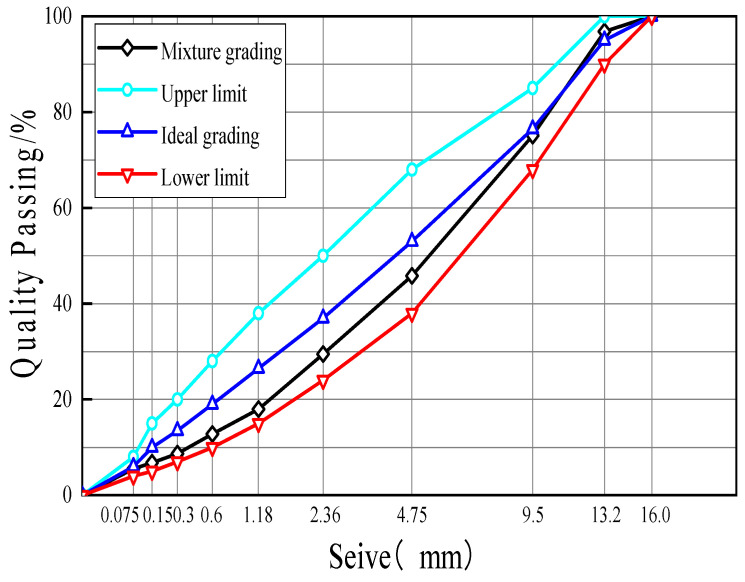
Gradation curve.

The dense-graded gradation of AC-13, featuring a nominal maximum aggregate size (NMAS) of 13.2 mm, was created using the Marshall method. The gradation curve is shown in Figure 3. BF, GF, and PF were blended into the asphalt mixture according to 0.1%, 0.2%, 0.3%, 0.4%, and 0.5% of the mass of the mixture to prepare a fiber-reinforced asphalt mixture.

Table 2 illustrates the optimum asphalt content of the asphalt mixture under different fiber types and content. The fiber content selected in this paper is from the previous research results [32].

The asphalt mastic powder-to-binder ratio prepared in this study was 1:1, and the fiber content in the asphalt mastic was set as 1%, 2%, 3%, 4%, and 5%. To ensure the uniformity of samples, a precision electric stirrer was used to prepare the fiber asphalt mastic. The process for preparing fiber asphalt mastic began by placing asphalt and mineral powder in an oven set at 175 °C for 2 h. After this, the mineral powder was added, and the mixture was stirred at a rotational speed of 2000 rpm for a duration of 5 min. Finally, the fiber was added to the asphalt mastic three times to decrease the agglomeration and was held for 10 min at 1000 rpm. In the process of sample preparation, three sets of parallel samples were prepared.

### 2.3. Test Methods

#### 2.3.1. Binder Bond Strength Test (BBS)

**Figure 4:**
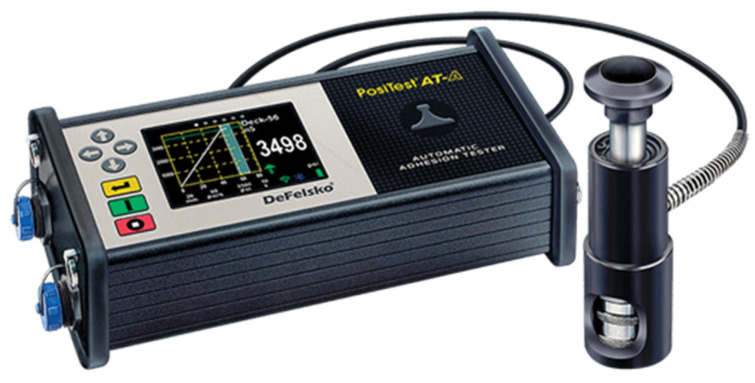
Test system.

BBS was conducted using an interfacial pullout test system. An automatic adhesion tester, a mineralogical stone plate, and a spindle are included in this system as shown in Figure 4. The technical parameters of the instrument are shown in Table 3. The basic principle is to apply the asphalt material to the surface of the aggregate or spindle and apply vertical tension to the spindle for pulling, and the interfacial strength between asphalt and aggregate can be reacted by tensile force or tensile strength.

The interfacial strength can be calculated by Equation (1).

(1) $$\sigma =\frac{4F}{\pi D^{2}}$$

where σ is the interfacial strength (MPa); F is the tensile force (kN); D is the inner diameter of the spindle (mm).

The testing steps are as follows:

(1) Heat the spindle in the oven at 170 °C for 1 h, and the substrate in the oven at 50 °C for 2 h.
(2) Place the silicone ring in the middle of the substrate to prevent the overflow of samples.
(3) Apply pressure to the center of the spindle for 5 min to make it fully bonded.
(4) Clean excess residual asphalt, and carry out the test after 2 h of heat preservation.

#### 2.3.2. Disk-Shaped Compact Tension Test (DCT)

The cracking performance of asphalt mixtures was tested using the DCT test method with a strain control method to ensure that the rate of opening displacement (CMOD) was 1 mm/min, and the test was terminated when the test stress level peaked and then recovered to 0.1 kN. This study investigates the effect of different fiber types (PF, GF, and BF), different fiber content (0%, 0.1%, 0.2%, 0.3%, 0.4%, and 0.5%), and aggregate (limestone and basalt) types on the cracking resistance of fiber-reinforced asphalt mixture.

Fracture energy (G_f_) can be used to evaluate the cracking resistance of asphalt mixtures, which can be calculated by Equation (2), and the envelope area of the LOAD–CMOD curve can be calculated by Equation (3).

(2) $$G_{f}=\frac{Area}{B\cdot \left(W-a\right)}$$

where G_f_ is fracture energy (Jm^2^); Area is the area of envelope of LOAD–CMOD curve (mm·kN); B is the thickness of the specimen (m); W-a is the initial ligament length of the specimen (m).

(3) $$Area=\sum_{i=1}^{n}\left(x_{i+1}-x_{i}\right)\cdot \left(y_{i}\right)+0.5\cdot \left(x_{i+1}-x_{i}\right)\cdot \left(y_{i+1}-y_{i}\right)$$

where Area is area of envelope of LOAD–CMOD curve (mm·kN); x is the opening displacement of the specimen (mm); y is the load (kN); n is the number of data points when the stress level peaks and then recovers to 0.1 kN.

#### 2.3.3. Interface Image Processing Method

**Figure 5:**
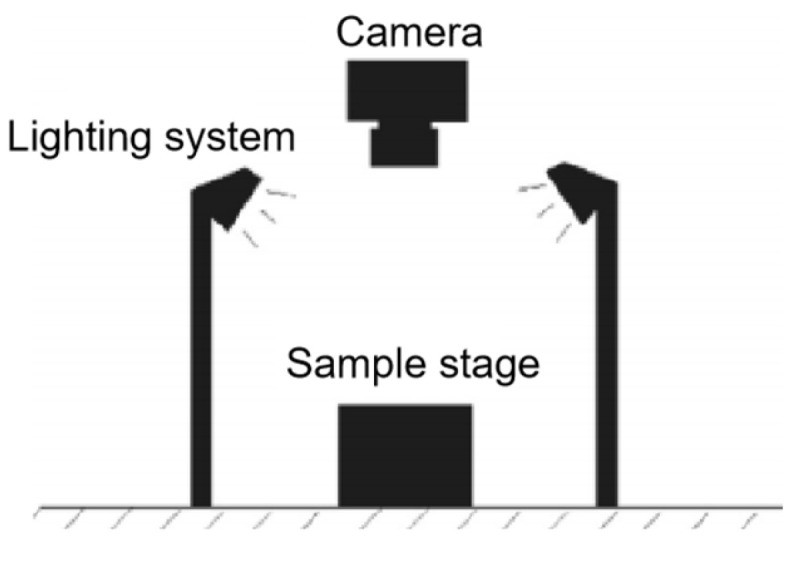
Imaging system.

In order to analyze the damage mode of the interface between asphalt mastic and aggregate after the binder bond strength test, a digital camera acquisition system was used to collect the images, as shown in Figure 5; with the help of the image analysis software (Image-Pro Plus V 7.0, IPP), the section images of the BBS-pulled specimen were analyzed, and the area of the asphalt mortar was extracted and bare aggregate area, indicating cohesion damage and adhesion damage, respectively.

The interface strength obtained by the test is composed of two parts: bonding strength (σ_a_) and cohesion strength (σ_c_). The adhesive damage area (S_a_) and cohesive damage area (S_c_) were obtained by image acquisition and recognition, and the adhesive strength and cohesive strength could be calculated by the formula [32].

#### 2.3.4. Moisture Infiltration Treatment Method

Moisture infiltration treatment of BBS and DIC samples was carried out according to the immersion Marshall test in the water stability test of the asphalt mixture. The specimens were immersed at 60 °C for 7 days. After the immersion, the samples were kept at 25 °C for 4 h, and then the BBS and DIC tests were carried out under the same experimental conditions.

## 3. Results and Discussion

### 3.1. Interfacial Strength of Fiber Asphalt Mastic and Aggregate

#### 3.1.1. Effect of Fiber and Aggregate on the Interfacial Strength

**Figure 6:**
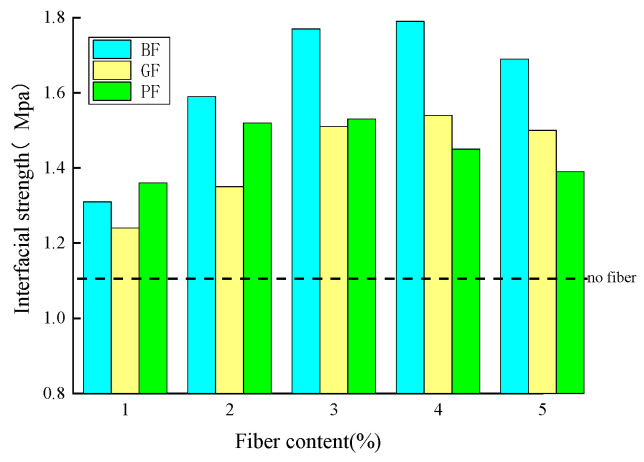
Effect of fiber content and type on the interfacial strength.

**Figure 7:**
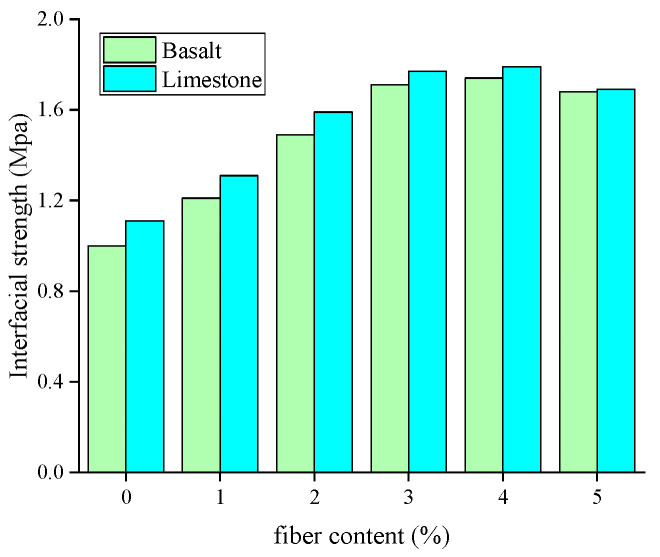
Effect of aggregate type on the interfacial strength.

Figure 6 shows the influence of two factors of fiber (fiber type and content) on the interfacial strength of fiber asphalt mastic and aggregate at 25 °C. It can be observed from Figure 7 that compared with the neat one, the addition of fibers can significantly enhance the interfacial strength. With the rise in fiber content, the interfacial strength of fiber asphalt mastic and aggregate increased significantly and then decreased slightly. With the fiber content reaching 3–4%, the improvement effect on the interfacial strength is the best, which increases by 30.6–61.3%. Furthermore, BF has the best effect on the enhancement of interfacial strength, followed by GF and PF. This may be due to BF having the best mechanics and being able to play a better bridging role.

Figure 7 illustrates the effect of aggregate type on the interfacial strength. The results show that the interfacial strength between asphalt mixture and limestone aggregate (LA) and basalt aggregate (BA) is 1.11 MPa and 1.00 MPa, respectively.

#### 3.1.2. Effect of Moisture Infiltration on Interfacial Strength

**Figure 8:**
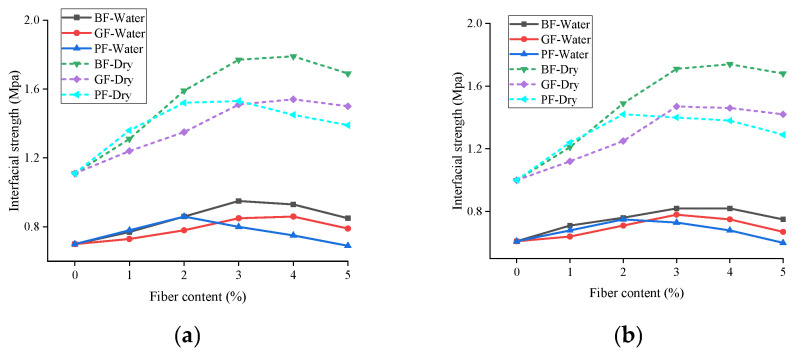
Interfacial strength under different environments: (**a**) LA; (**b**) BA.

**Figure 9:**
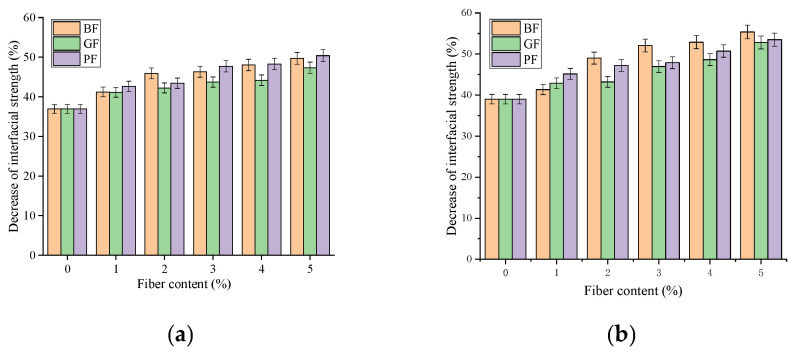
Decrease in interfacial strength after moisture infiltration: (**a**) LA; (**b**) BA.

Figure 8 illustrates the interfacial strength of asphalt mastic and aggregate under dry environment, and moisture infiltration; the reduction of interfacial strength after moisture infiltration is shown in Figure 9.

It can be observed from Figure 8 that in a dry environment, the interface strength is 1.11–1.77 MPa; in a water environment, the interface strength is 0.7–0.95 MPa, and the interfacial strength decreases significantly after the influence of moisture infiltration. Compared with the dry state, with the rise in fiber content, the reduction of interfacial strength after moisture infiltration increased, up to 53.49%. This is due to water penetrating from the edge of the asphalt film to the inside of the aggregate and mastic, damaging the bonded interface and reducing the interfacial strength. Taking the limestone aggregate as an example, the interfacial strength of BF, GF, and PF asphalt mastic and aggregate decreased 41.22–49.70%, 41.13–47.33%, and 42.65–50.36%, respectively, after moisture infiltration. This indicates that the moisture infiltration had the greatest negative effect on the interfacial strength between PF asphalt mastic and aggregate. Furthermore, it can be seen that under the influence of moisture infiltration, the interfacial strength of limestone aggregate and asphalt decreases by 36.94–50.36%, and the interfacial strength of basalt aggregate and asphalt decreases by 39.00–53.49%, with a greater decline. This is mainly because higher SiO_2_ content will accelerate the occurrence of water damage in asphalt mixture [33], so moisture infiltration has little effect on the interfacial strength of limestone aggregate and asphalt.

### 3.2. Effect of Moisture Infiltration on Failure Mode of Fiber Asphalt Mastic and Aggregate Interface

**Figure 10:**
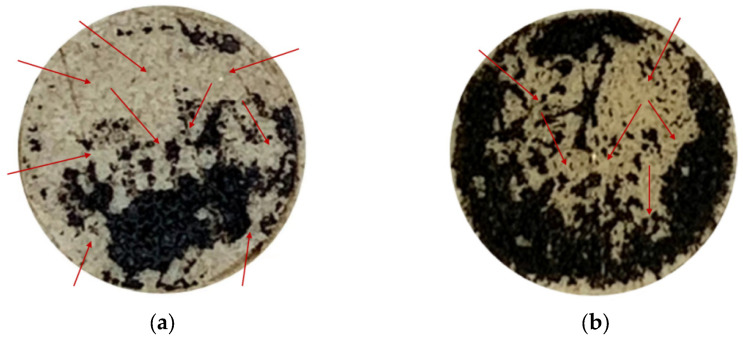
Images of interface failure between asphalt mastic and aggregate after moisture infiltration: (**a**) Basalt aggregate; and (**b**) limestone aggregate.

Figure 10 shows the images of interface failure between asphalt mastic and aggregate after moisture infiltration. It can be seen that interface failure is a comprehensive mode, including both adhesive failure and cohesive failure, but the two kinds of aggregate interface failure patterns are different. Specifically, for basalt aggregate, the interface failure is from the edge to the center; on the contrary, limestone aggregate is from the center to the edge. This is mainly due to the difference in the path of moisture infiltration. The red arrow represents the path of moisture infiltration. It can be observed from Figure 10 that there are many weak points at the edge of the interface between asphalt mastic and basalt aggregate; moisture will invade weak points at the edge of the interface and then gradually invade the inside of the interface, replacing the asphalt film on the surface of the aggregate, resulting in extensive spalling of fiber asphalt mastic on the surface of the aggregate. On the contrary, the boundary of the asphalt mastic and limestone aggregate interface has fewer weak points and fewer moisture infiltration points, resulting in more complete edge adhesion.

**Figure 11:**
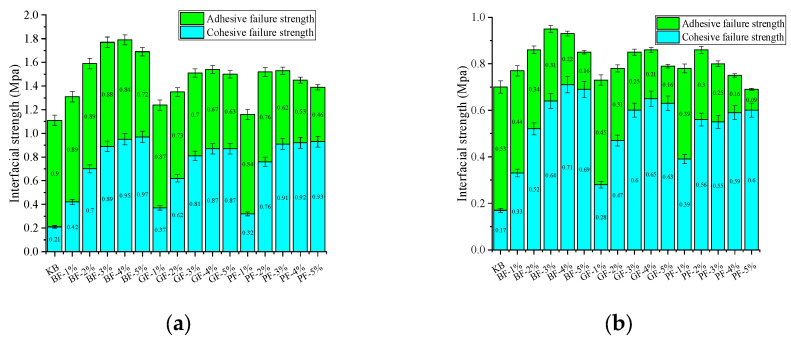
Interfacial strength:(**a**) Dry environment; (**b**) moisture infiltration.

Figure 11 compares the interfacial interaction strength under a dry environment and moisture infiltration conditions. As can be seen from the picture, regardless of adhesive strength or cohesive strength, the interfacial strength in a dry environment is always higher than moisture infiltration. This is due to the gradual invasion of water, replacing the asphalt membrane on the surface of the aggregate so that the interface strength is reduced.

**Figure 12:**
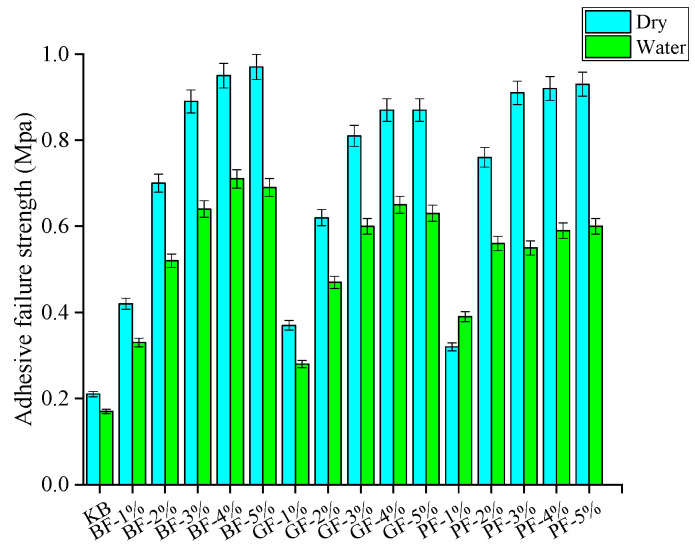
Adhesion strength.

**Figure 13:**
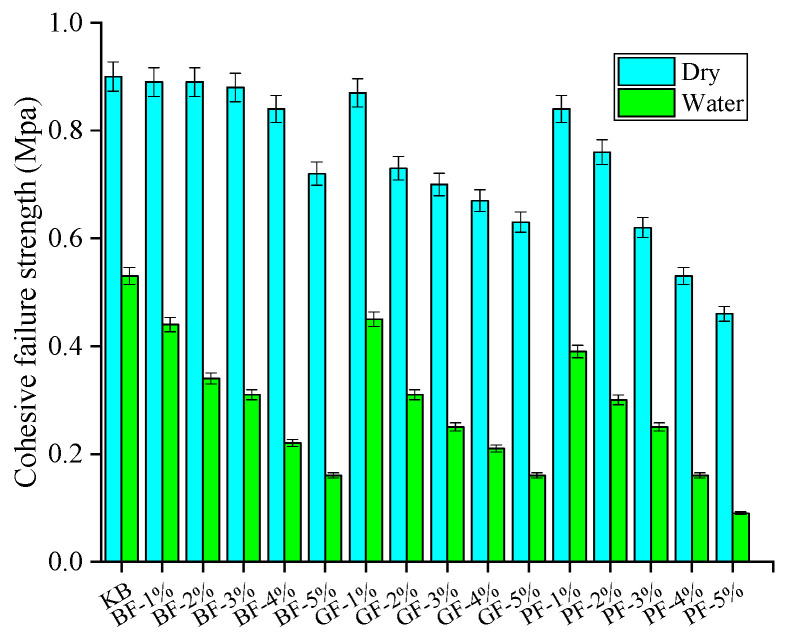
Cohesive strength.

Figure 12 and Figure 13 illustrate the comparative results of adhesive strength and cohesive strength under different circumstances, respectively. After infiltration, the interfacial cohesive strength decreased from 19.05% to 39.56%, and the interfacial adhesive strength decreased from 41.11% to 80.43%. It can be seen that the main reason for the decrease in interaction strength under the influence of moisture infiltration is the decrease in adhesion strength. This is because the addition of fiber can not only improve the cohesive strength but also slightly reduce the adhesive strength between the interfaces. However, as the water gradually invades and replaces the asphalt membrane on the aggregate surface in the moisture infiltration, the adhesive strength between the interfaces is further weakened and finally leads to a rapid decline in the interaction strength between the fiber asphalt mastic-aggregate interface after infiltration.

### 3.3. Cracking Resistance of Fiber-Reinforced Asphalt Mixture

#### 3.3.1. Effect of Fiber and Aggregate on Cracking Resistance

**Figure 14:**
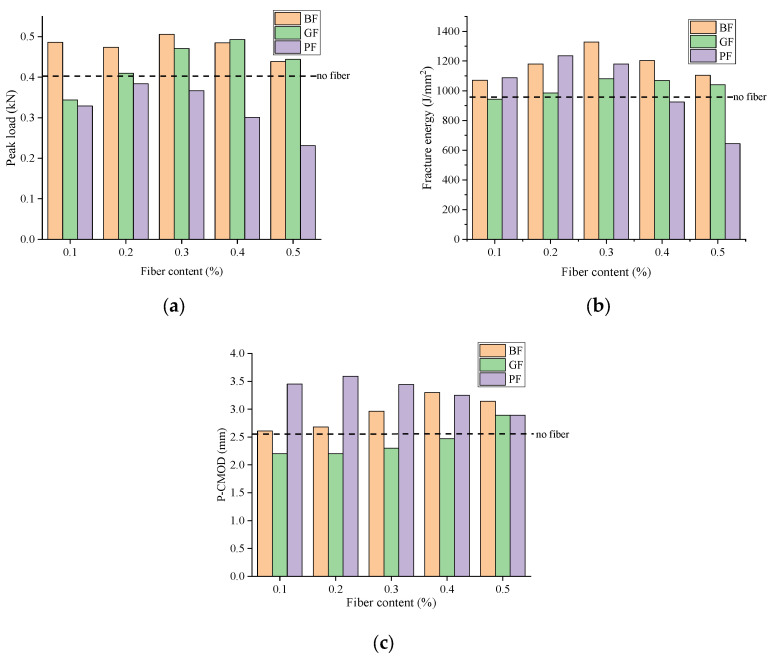
Effect of fiber content and type on cracking resistance: (**a**) Peak load; (**b**) fracture energy; and (**c**) P-CMOD.

Figure 14 illustrates the effect of fiber content and type on the cracking resistance of fiber-reinforced asphalt mixture. As shown in Figure 14a,b, when the fiber content increased from 0.1% to 0.5%, the peak load of the fiber asphalt mixture first increased and then decreased, and the fracture energy also changed in this trend; the values were the largest, which were 0.506 kN and 1327.5 J/m^2^, respectively. Compared with the neat one, the values are increased by 24.02% and 35.32%, respectively, indicating that the medium fiber content has the best effect on the peak load and fracture energy of the fiber asphalt mixture. It can be seen from Figure 14c that the P-CMOD of the mixture increases with the increase of BF and GF fiber content, while the P-CMOD of the PF asphalt mixture increases first and then decreases with the increase of fiber content.

**Figure 15:**
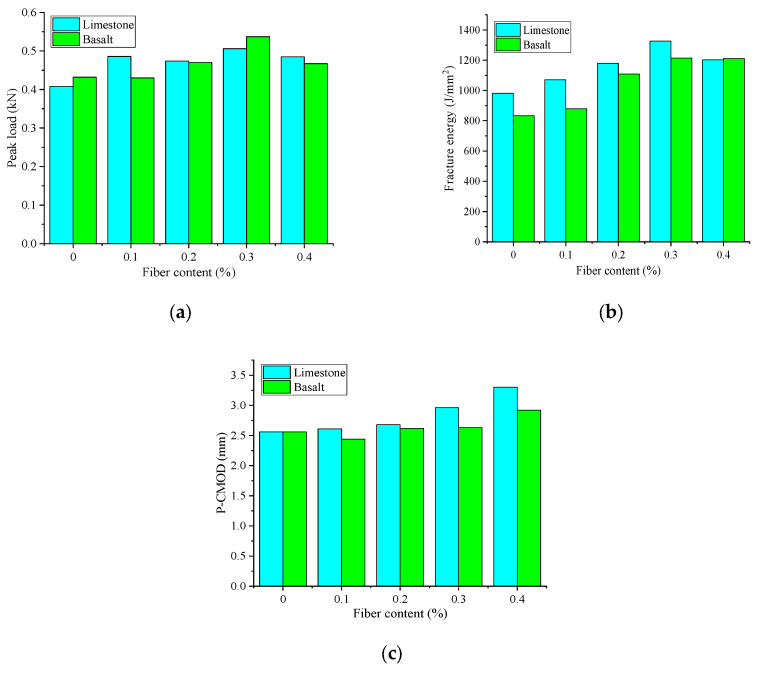
Result of aggregate type on cracking resistance: (**a**) Peak load; (**b**) fracture energy; and (**c**) P-CMOD.

Figure 15 illustrates the impact of aggregate type on the cracking resistance of fiber-reinforced asphalt mixture. As can be seen from Figure 15, the peak load of fiber-reinforced asphalt mixture with limestone aggregate and basalt aggregate is 0.408–0.506 kN and 0.43–0.537 kN, respectively, and the fracture energy is 981–1327.5 J/m^2^ and 833–1214.5 J/m^2^, respectively. P-CMOD is 2.56–3.143 mm and 2.44–2.92 mm, respectively. It can be seen that limestone aggregate asphalt mixture is superior to basalt aggregate in the experimental results.

#### 3.3.2. Effect of Moisture Infiltration on Cracking Resistance

**Figure 16:**
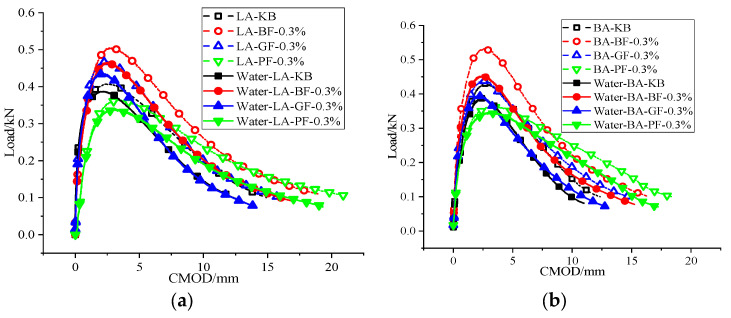
DCT test LOAD–CMOD curve: (**a**) LA; and (**b**) BA.

**Figure 17:**
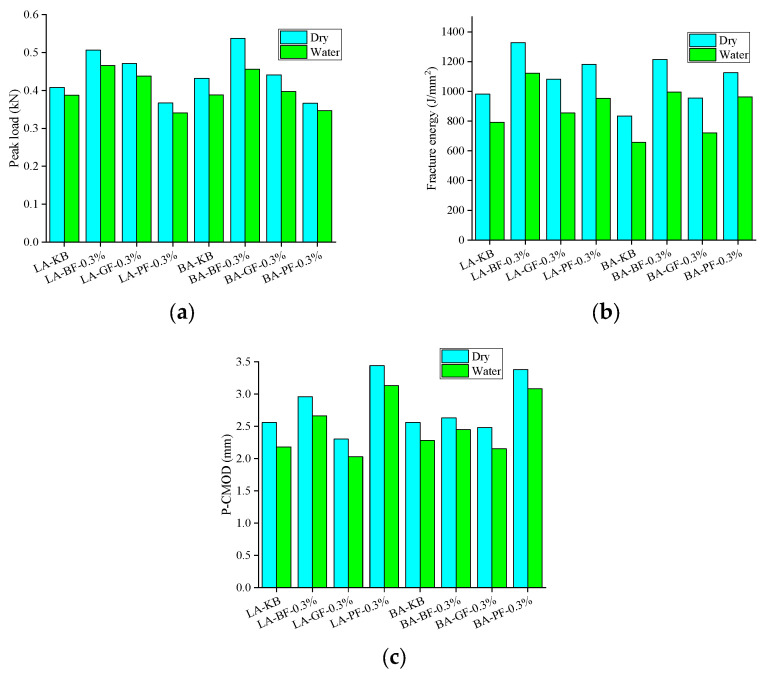
Effect of moisture infiltration on cracking resistance: (**a**) Peak load; (**b**) fracture energy; and (**c**) P-CMOD.

Figure 16 and Figure 17 show the CMOD-load curve, peak load, fracture energy, and P-CMOD results of the DCT test under wet and dry conditions. It can be observed that the peak load, fracture energy, and P-CMOD of asphalt mixture decrease to some extent after moisture infiltration. Furthermore, the decrease range of peak load is 5.15–15.08%, the decrease range of fracture energy is 14.57–24.65%, and the decrease range of P-CMOD is 7.02–15.01%. It can be seen that the decreased range of fracture energy is the largest, indicating that moisture infiltration has the most significant effect on the fracture energy of the asphalt mixture. The explanation could be that the load reduction rate of the asphalt mixture after reaching the peak load is significantly accelerated after moisture infiltration, resulting in a significant decrease in fracture energy. In the process of the initial load gradually rising to the peak load, the coincidence degree of the test curve before and after moisture infiltration is high, so the decreased range of peak load and P-CMOD after moisture infiltration is obviously smaller than the decreased range of fracture energy.

#### 3.3.3. Effect of Moisture Infiltration on Failure Mode of the Mixture

**Figure 18:**
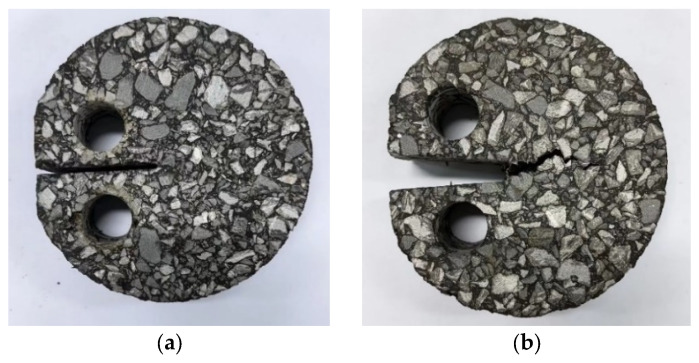
DCT test specimen; (**a**) Before test; (**b**) after test.

Figure 18 shows the state of the DCT test specimen after the test. It can be observed that during the test, cracks develop from the tip of the initial precut seam to the other end of the specimen. When the tensile load applied to the specimen is less than 0.1 kN, the test is terminated. At this time, cracks did not completely penetrate the asphalt mixture specimen, as shown in Figure 18b.

**Figure 19:**
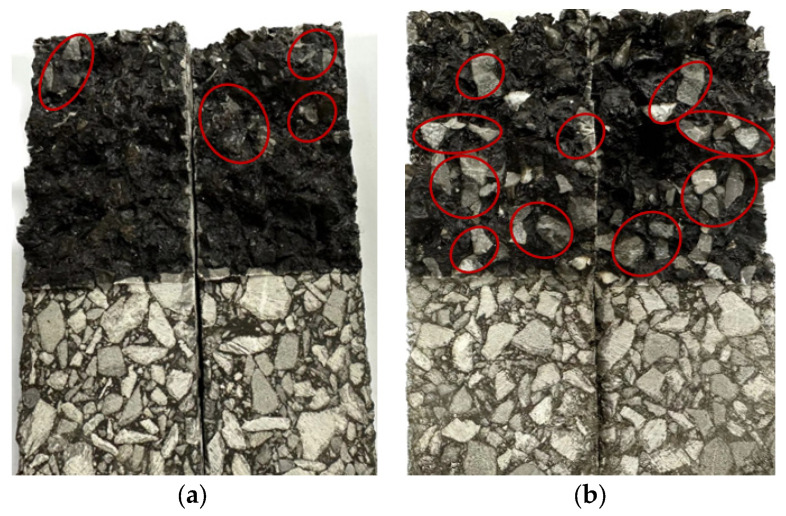

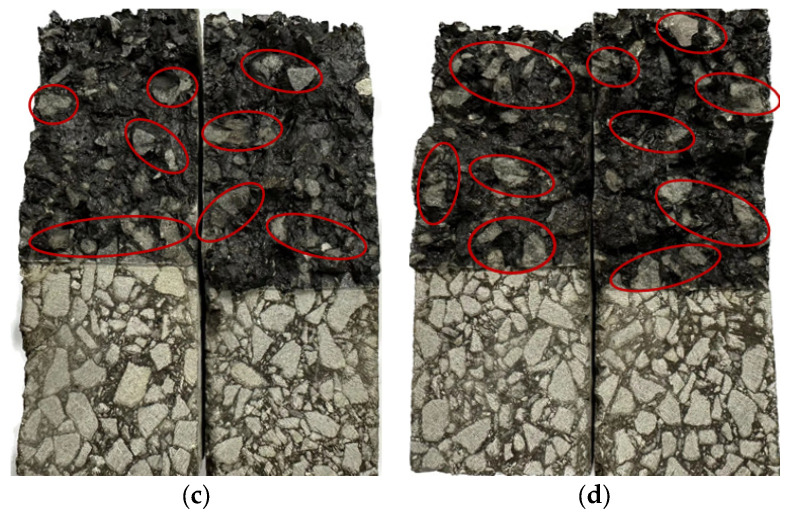
Fracture morphology of the specimen: (**a**) LA; (**b**) LA-Water; (**c**) BA; and (**d**) BA-Water.

In order to further observe the fracture morphology of the fiber asphalt mixture specimen after the DCT test, the load was continuously applied until the specimen was completely broken and destroyed, and then the fracture morphology was collected, as shown in Figure 19. It is clear that the exposed aggregate area in the fracture of limestone aggregate specimen without moisture infiltration in Figure 19a is small, while the exposed aggregate area in the fracture of limestone aggregate specimen with moisture infiltration in Figure 19b is significantly increased. This indicates that the moisture infiltration causes the interface failure between the aggregate and the asphalt mastic. Under the action of tension, the phenomenon of stripping occurs between the asphalt mastic and the aggregate, which leads to a decrease in cracking resistance [35].

Comparing Figure 19a,c, it can be seen that the exposed aggregate of limestone aggregate specimen is significantly less than that of basalt in fracture before moisture infiltration. This shows that the combination of limestone aggregate and asphalt mastic is generally better than that of basalt aggregate. Furthermore, after moisture infiltration, the exposed aggregate in the fracture of the basalt aggregate specimen increased significantly and spread throughout the fracture, which also explains the reason why the cracking resistance of the basalt aggregate asphalt mixture decreased significantly after being subjected to moisture infiltration.

#### 3.3.4. Correlation Analysis of Interfacial Properties and Cracking Resistance of Mixture Under Moisture Infiltration

Previous studies [32] showed that the correlation between the interfacial strength and the cracking resistance indexes is high in the dry state. Especially, the interfacial strength has the greatest influence on the fracture energy and P-CMOD during the cracking of asphalt mixtures. In order to further investigate the influence of the moisture infiltration on the correlation between the interfacial strength and the cracking resistance, the grey correlation index of interfacial strength and peak load, fracture energy, and P-MOD were calculated, respectively.

**Figure 20:**
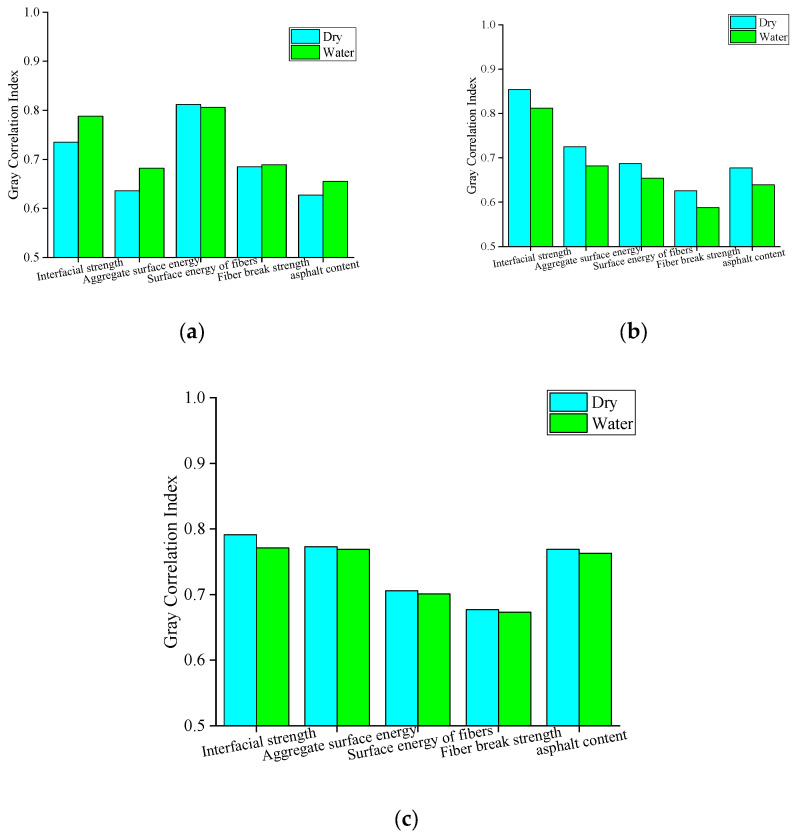
Gray correlation index; (**a**) Peak loads; (**b**) fracture energy; and (**c**) P-CMOD.

Six samples with a fiber content of 0.3% were selected; Figure 20 shows the correlation index of multiple factors on peak load, fracture energy, and P-CMOD under different conditions. As can be seen from Figure 20, the correlation index between interfacial strength and peak load after being affected by moisture infiltration is 0.788. Compared with the dry state, the value increased by 7.21%. This indicates that the influence of interface strength on the peak load of asphalt mixture is increased to some extent under the influence of moisture infiltration. After moisture infiltration, the correlation index between interfacial strength and fracture energy is 0.812, which decreases by 4.92% compared with the dry state. However, the correlation between interfacial strength and fracture energy is much higher than that of other influencing factors, whether in the dry state or immersed state, indicating that even under the influence of moisture infiltration, interfacial strength is still one of the decisive factors affecting the fracture energy of asphalt mixture. After moisture infiltration, the correlation index of interfacial strength, surface energy of aggregate, asphalt content, and P-CMOD are 0.771, 0.769, and 0.763, respectively, and the values are very close. This indicates that the interfacial strength, surface energy of aggregate, and asphalt content have a great influence on the speed of the asphalt mixture reaching peak load after moisture infiltration.

## 4. Conclusions

In this paper, different fiber contents and types were selected to prepare the fiber-reinforced asphalt mastic and asphalt mixture. Bond Strength Test (BBS), Disk-Shaped Compact Tension test (DCT), and interfacial image processing were used to investigate the effects of moisture infiltration on the interfacial characteristics of fiber asphalt mastic-aggregate and the cracking resistance of the mixture. The main conclusions are as follows:

(1) The addition of fibers could enhance fiber asphalt mastic-aggregate interfacial strength. Among the three kinds of fibers, basalt fiber has the most effective effect on interfacial strength; compared with basalt aggregate, limestone aggregate has a greater impact on interfacial strength.
(2) Under the influence of moisture infiltration, the interfacial strength between fiber asphalt mastic and aggregate shows a significant decrease, especially in the interfacial properties of PF asphalt mastic and aggregate. Compared to basalt aggregate, limestone aggregate is able to resist moisture infiltration more effectively, thus reducing the decline in interfacial strength.
(3) The addition of fibers can effectively improve the cracking resistance of asphalt mixture. BF can effectively increase the peak load and fracture energy, and PF can effectively increase the P-CMOD.
(4) The interface between asphalt mastic and aggregate is more prone to spalling after being subjected to moisture infiltration, resulting in a decrease in cracking resistance. Compared with basalt aggregate, the cracking resistance of limestone aggregate asphalt mixture is not significantly reduced under the influence of moisture infiltration. Limestone aggregates have better resistance to moisture infiltration, so they can be used in rainy regions.
(5) Compared with the dry environment, after moisture infiltration, the correlation index between interfacial strength and fracture energy is much higher than other influencing factors. The interfacial strength is still an important factor affecting the fracture energy of the asphalt mixture even though it is affected by moisture infiltration.
(6) The mechanism of asphalt peeling from the aggregate surface under moisture infiltration can be studied by means of analytical dynamic simulation.

## Figures and Tables

**Table 1:** Fiber oil absorption rate.

Fiber Type	Basalt Fiber	Glass Fiber	Polyester Fiber
Tensile strength MPa	2561	2318	979
Oil absorption rate/times	1.04	0.5	3.43

**Table 2:** Optimum asphalt content of asphalt mixture under different fiber types and content.

Fiber Type	Fiber Content/%				
	0.1	0.2	0.3	0.4	0.5
BF	4.9	5.0	5.1	5.2	5.2
GF	4.9	5.0	5.1	5.2	5.2
PF	5.0	5.1	5.2	5.3	5.3

**Table 3:** Technical parameter.

Spindle Diameter/mm	Resolution/MPa	Accuracy	Range/MPa
20	0.01	±1%	0–20

## Data Availability

The original contributions presented in this study are included in the article. Further inquiries can be directed to the corresponding author.
